# Isoeugenol is a selective potentiator of camptothecin cytotoxicity in vertebrate cells lacking TDP1

**DOI:** 10.1038/srep26626

**Published:** 2016-05-25

**Authors:** Waheba Elsayed, Lamia El-Shafie, Mohamed K. Hassan, Mohamed A. Farag, Sherif F. El-Khamisy

**Affiliations:** 1 Center for Genomics, Helmy Institute for Medical Sciences, Zewail City of Science and Technology, Giza, Egypt; 2 Biotechnology Dept., Faculty of Science, Port Said University, Egypt; 3 Department of Pharamcognosy, Faculty of Pharmacy, Cairo University, Cairo, 11562, Egypt; 4 Krebs Institute, University of Sheffield, Sheffield, S10 2TN, UK

## Abstract

Camptothecin (CPT), a topoisomerase I (TOP1) inhibitor, exhibits anti-tumor activity against a wide range of tumors. Redundancy of TOP1-mediated repair mechanisms is a major challenge facing the efficiency of TOP1-targetting therapies. This study aims to uncover new TOP1 targeting approaches utilising a selection of natural compounds in the presence or absence of tyrosyl DNA phosphodiesterase I (TDP1); a key TOP1-mediated protein-linked DNA break (PDB) repair enzyme. We identify, isoeugenol, a phenolic ether found in plant essential oils, as a potentiator of CPT cytotoxicity in Tdp1 deficient but not proficient cells. Consistent with our cellular data, isoeugenol did not inhibit Tdp1 enzymatic activity *in vitro* nor it sensitized cells to the PARP1 inhibitor olaparib. However, biochemical analyses suggest that isoeugenol inhibits TDP2 catalytic activity; a pathway that can compensate for the absence of TDP1. Consistent with this, isoeugenol exacerbated etoposide-induced cytotoxicity, which generates TOP2-mediated PDBs for which TDP2 is required for processing. Together, these findings identify isoeugenol as a potential lead compound for developing TDP2 inhibitors and encourage structure-activity relationship studies to shed more light on its utility in drug discovery programs.

Camptothecin (CPT), a quinoline alkaloid isolated from the wood and bark of the Chinese tree *Camptotheca acuminata*, is a well-known topoisomerase I (TOP1) inhibitor^1^ that was first isolated and identified as an anti-cancer agent in 1966^2^. TOP1 functions to relax supercoils by creating a nick in the DNA strand and forming a covalent bond between its tyrosyl residue and a 3′ DNA phosphate. An intermediate structure known as TOP1 cleavage complex (TOP1cc) is formed through cleavage, followed by religation of the DNA strand. CPT acts as a cellular poison by reversibly trapping TOP1ccs onto the DNA. TOP1ccs are normally transient; however collision of replication or transcription machineries with TOP1cc converts them to irreversible protein-linked DNA single-strand breaks (SSBs) or double-strand breaks (DSBs)^3^. Tyrosyl DNA phosphodiesterase I (TDP1) catalyzes the hydrolysis of the tyrosyl-3′ phosphate linkage formed between TOP1 and DNA, allowing the rejoining of the DNA strand break and hence help cells to overcome CPT-induced cytotoxicity. Mutations of *TDP1* lead to the autosomal recessive disease SCAN-1^4^. Cell lines derived from patients with SCAN-1 accumulate TOP1-mediated protein-linked DNA breaks (PDBs) and were found to be hypersensitive to CPT^5^.

Owing to CPT’s severe toxicity, other semisynthetic analogues have been developed, namely irinotecan and topotecan, which are currently used alone or in combination with other drugs as chemotherapeutic agents^6^. Irinotecan, also known as CPT-11, is approved for colon cancer treatment, whereas topotecan is used for the treatment of ovarian cancer. CPT-11 is mainly used for the treatment of metastatic colon cancer as part of a combination regimen alongside 5-fluorouracil. Several studies have demonstrated that combined therapy of two or more drugs against cancer cells promotes synergism, enhances therapeutic effectiveness, suppresses drug resistance, and reduces the drug side effects^7^,^8^. Functional redundancy in repairing TOP1-mediated PDBs is a potential cause for resistance to TOP1-based chemotherapy strategies. Herein, we investigated the cytotoxic effects of 41 natural compounds in combination with CPT in the presence or absence of TDP1, with the goal of unraveling novel approaches to target TOP1.

We assembled a library of natural derived chemicals that are isolated from either plant or animal origin. Natural products provide a vast resource for drug discovery research. For example, in 1971 and as a part of a National Cancer Institute program, paclitaxel was identified as the active constituent from a crude extract of the bark of *Taxus brevifolia*^9^. Paclitaxel is the first taxane to enter clinical trials as a chemotherapeutic agent and is used against ovarian, breast, and lung cancers^10^. *Ginkgo biloba* extract 761 (EGb761) is another natural compound extracted from *Ginkgo biloba* leaves^11^ that was proposed to have anti-angiogenic and antioxidant activities.

Our screen yielded a hit compound that appears to be a promising lead as a tyrosyl DNA phosphodiesterase II (TDP2) inhibitor. Like TDP1, TDP2 is a tyrosyl DNA phosphodiesterase but that catalyzes the cleavage of 5′-tyrosyl bonds that are present between topoisomerase II (TOP2) and the DNA^3^. It is known that both enzymes while structurally different, can serve as back up for each other in circumstances where one is lacking, but with much less efficiency than with their preferred substrate^12^.

## Results

### Establishment of sub-lethal concentrations of the library compounds

**Figure 1:**
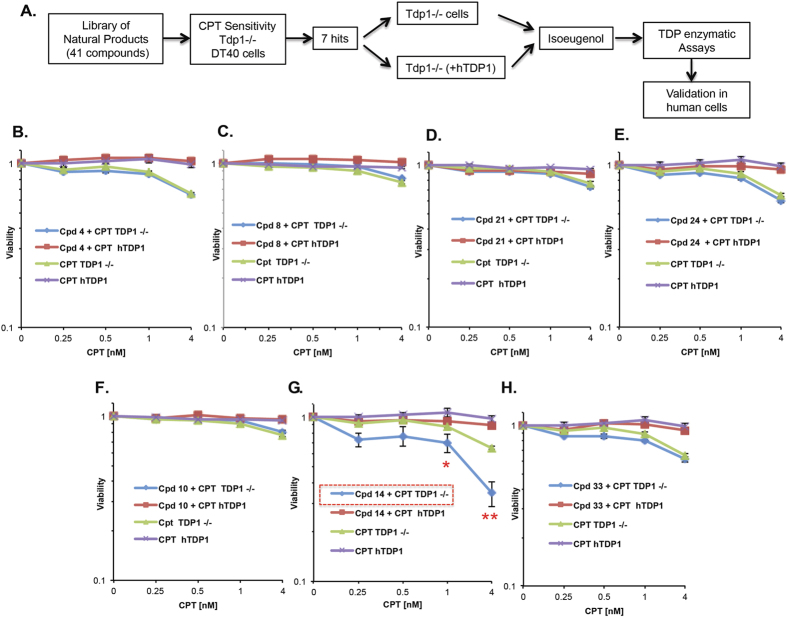
Isoeugenol potentiates the cytotoxic effect of CPT in a TDP1-dependent manner. (**A**) A diagram summarizing the experimental strategy employed in this study. (**B–H**) Viability of Tdp1−/− and Tdp1−/− DT40 cells complemented with hTDP1 was compared using the indicated doses of camptothecin (CPT) in presence or absence of sub-lethal concentrations of compounds 4, 8, 10, 14, 21, 24 and 33 as indicated in Table 1. Results are presented on a semi-log scale and represent the average of 3 biological replicates ± s.e.m. Asterisks denote statistical difference (p < 0.001, 4 nM; p < 0.05, 1 nM) using t-test.

A selection of 41 diverse natural products most of which occur in foods or dietary supplements were subjected to a series of cellular and biochemical experiments to identify potential hits to improve TOP1 targeting therapy (Fig. 1A). The large therapeutic index reported for these chemicals is encouraging in terms of developing future and safe drugs for treatment of cancer. The library is comprised of molecules belonging to different natural products classes viz. alkaloids, flavonoids, saponins, terpenes, and phenylpropanoids. To determine sub-lethal doses, the cytotoxic effect of each compound was examined at six different concentrations (0.01, 0.1, 1, 5, 10, and 50 μM) in Tdp1−/− DT40 cells (Supplementary Fig. 1). The viability assay used in this study utilizes a blue dye, resazurin, that when encountered with viable cells, is reduced to the highly fluorescent red dye resorufin^13^. The cytotoxicity, therefore, is measured as fluorescence intensity quantified by a plate reader. The selected sub-lethal doses (Table 1) were then used for the primary screen in combination with a single lethal and three sub-lethal doses of CPT (4, 1, 0.5, 0.25 nM) in the same cellular model (Supplementary Figs 2 and 3). The combination of any of the compounds 4, 8, 10, 14, 21, 24, or 33 with CPT gave the highest levels of cytotoxicity (viability inhibition) ranged from 81% for compound 8 to 47% for compound 24, at the highest concentration of CPT.

### Examination of the TOP1-mediated cytotoxic effect in the presence or absence of human TDP1

Seven compounds including prunin, isoquercetin, 2,4,4′ trihydroxy chalcone, isoeugenol, xanthohumol, harmine, and thymol (4, 8, 10, 14, 21, 24, 33, resp.) that showed the highest synergistically lethal effect with CPT in Tdp1−/− cells were selected for further screening by comparing their effect on chicken DT40 Tdp1−/− and an isogeneic control stably expressing human TDP1 (hTDP1) (Fig. 1 and Supplementary Fig. 4). The intensity of fluorescence was normalized to a DMSO control (same volume as that of the highest concentration of combined drugs) and the results were obtained from three biological replicates. All tested compounds, except compound 14, gave the same effect on both cell lines (Tdp1−/− and Tdp1−/− complemented with hTDP1). Isoeugenol annotated as compound 14 revealed the highest, reproducible cytotoxic effect (p < 0.001, 4 nM; p < 0.05, 1 nM) when combined with CPT on Tdp1−/− cells but not on cells complemented with human TDP1, wherein the viability dropped from 65.5% (CPT alone) to 34.6% (CPT plus isoeugenol) at 4 nM CPT, and a reduction of ~18.2% at 1 nM CPT (Fig. 1G and Supplementary Fig. 4F). These results indicate that isoeugenol (4-propenyl-2-methoxyphenol), at 1 nM, does not exhibit a cytotoxic effect as a single agent, but rather holds a synergistic effect when combined with CPT in a TDP1-dependent manner. This was not an epiphenomenon of DT40 cells since isoeugenol also sensitized TDP1 depleted human MCF-7 cells to CPT, although the effect was much less apparent than DT40 cells (Supplementary Fig. 5).

### Isoeugenol does not act through the PARP1/TDP1 axis

**Figure 2:**
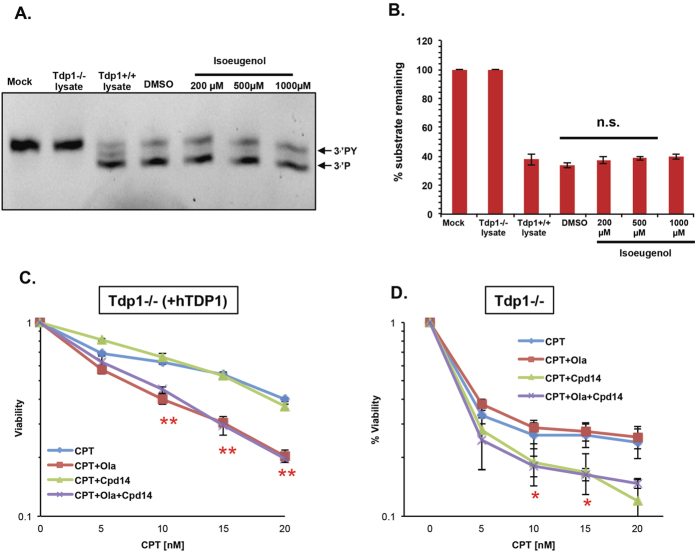
Isoeugenol does not inhibit TDP1 catalytic activity. **(A)** *In vitro* assay assessing the inhibitory effect of isoeugenol on TDP1. Isoeugenol concentrations of 200 μM, 500 μM, and 1000 μM were incubated with the 3-phosphotyrosine substrate and DT40 cell lysate (14 μg). Reaction mixture was fractionated on denaturing PAGE and analysed by a ChemiDoc™ MP Imaging System. Arrows point at the position of substrate (PY) and cleaved product (P). **(B)** Bands were quantified using ChemiDoc™ MP Imaging System software and the average % substrate remaining of three independent experiments ± s.e.m. is shown. **(C,D)** Viability of Tdp1−/− DT40 cells complemented with hTdp1 or Tdp1−/− DT40 cells following incubation with increasing concentrations of CPT alone or in the presence of the PARP inhibitor olaparib (0.1 μM) with and without isoeugenol (50 μM). Results are presented on a semi-log scale and represent the average of 3 biological replicates ± s.e.m. Asterisks denote statistical difference (hTDP1: p < 0.01, 10 nM and 15 nM; p < 0.001, 20 nM; Tdp1−/−: p < 0.05, 10 nM and 15 nM using t-test).

This data suggest that isoeugenol sensitizes cells to CPT only if TDP1 is absent, and that it may suppress TDP1 parallel pathways for repairing TOP1-induced PDBs. To further ascertain that the observed effects are independent of TDP1, we next examined the effect of isoeugenol on TDP1 catalytic activity using an *in vitro* biochemical assay employing a 13-mer oligonucleotide Cy5.5-labelled substrate containing a 3′-phosphotyrosine modification. TDP1, in the reaction mixture, cleaves tyrosine from the substrate allowing it to move faster and further through the gel, giving a slightly shifted band. As a positive control, total cell lysate from wild-type DT40 cells was used as a source of TDP1 while Tdp1−/− cell lysate was used as a negative control. Different concentrations of isoeugenol (200 μM, 500 μM, and 1000 μM) were incubated with the 3′-phosphotyrosine substrate along with the cell lysates and reaction products were fractionated by denaturing PAGE and analysed by fluorescence imaging. The addition of isoeugenol to the reaction did not inhibit the conversion of 3′-PY substrate to 3′-P (indicated by arrows) even at the highest concentration (1000 μM), confirming that isoeugenol does not inhibit TDP1 activity (Fig. 2A,B). We reasoned that if isoeugenol potentiates CPT cytotoxicity independently of TDP1 one would predict that co-addition of PARP1 inhibitors would be epistatic to isoeugenol, since PARP1 and TDP1 are working together in the same pathway for TOP-PDB repair. To test this hypothesis, we compared the viability of Tdp1−/− cells and controls following CPT treatment in presence and absence of isoeugenol and the FDA approved PARP inhibitor olaparib, either separately or in combination. Addition of olaparib to TDP1 proficient cells led to a marked increase in CPT cytotoxicity (Fig. 2C, p < 0.01). Importantly, co-addition of isoeugenol and olaparib did not further sensitise cells to CPT than addition of olaparib alone (Fig. 2C). In contrast to Tdp1 proficient cells, addition of olaparib to Tdp1−/− cells did not result in additional sensitisation, confirming that TDP1 and PARP1 operate in the same pathway (Fig. 2D). Notably, whilst addition of isoeugenol alone sensitized Tdp1−/− cells to CPT (p < 0.05), co-addition of isoeugenol did not. Together, we conclude from these findings that isoeugenol potentiates CPT cytotoxicity independently of the TDP1/PARP1 axis.

### Isoeugenol does not increase single-strand break (SSB) accumulation in CPT-treated cells

**Figure 3:**
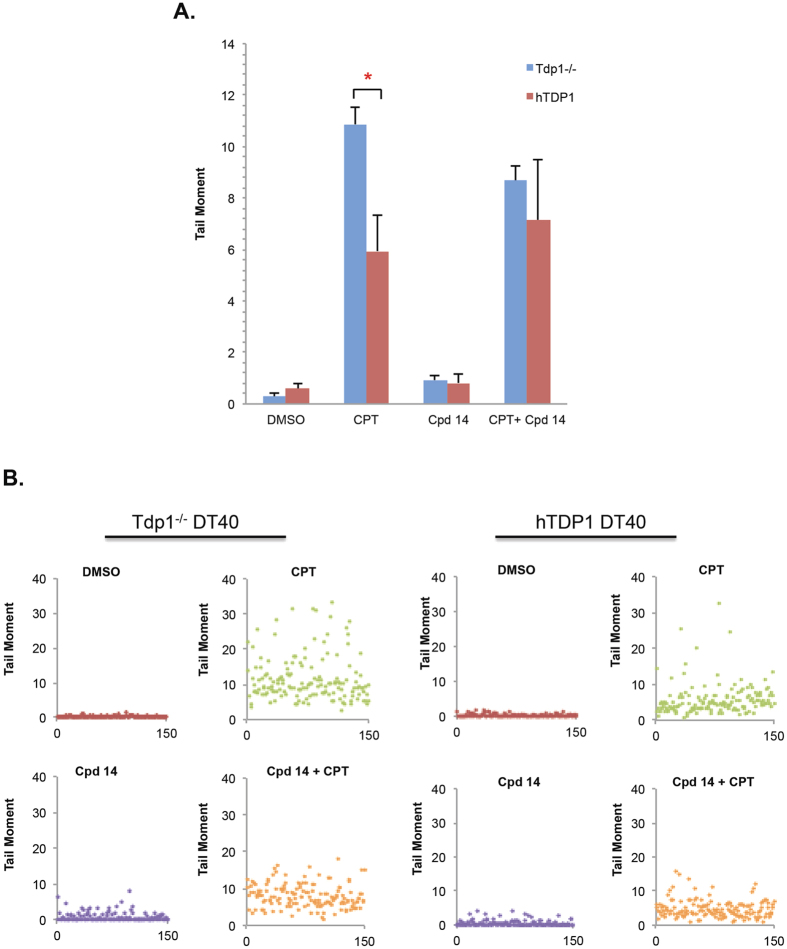
Isoeugenol does not increase SSB accumulation in CPT- treated cells. **(A)** The indicated cell lines were incubated with 50 μM CPT for 30 min or 50 μM isoeugenol for 48 h or both at 37 °C. DNA strand breakage was quantified using alkaline comet assays. **(B)** Representative scatter plots of 150 cells (cells numbered from 0 to 150) of CPT-treated or untreated cells with and without isoeugenol. Average tail moments from 150 cells per sample were measured, and data are the average of three independent biological replicates ± s.e.m. Asterisk denotes statistical difference (P < 0.05; t-test) between CPT TDP1−/− and CPT hTDP1 cells.

It is known that TDP1 deficient cells accumulate higher levels of DNA single-strand breaks (SSBs) in the presence of TOP1 inhibitors than controls^14^,^15^,^16^,^17^. We therefore examined if isoeugenol would increase the level of DNA strand breaks observed in Tdp1−/− DT40 cells, using alkaline single-cell gel electrophoresis (comet assays). As expected, the lack of TDP1 caused a CPT-dependent increase in SSB accumulation (P < 0.05); yet, isoeugenol did not potentiate such effect neither alone nor in combination with CPT (Fig. 3).

### Isoeugenol inhibits TDP2 catalytic activity *in vitro* and potentiates the cytotoxic effect of etoposide *in vivo*

**Figure 4:**
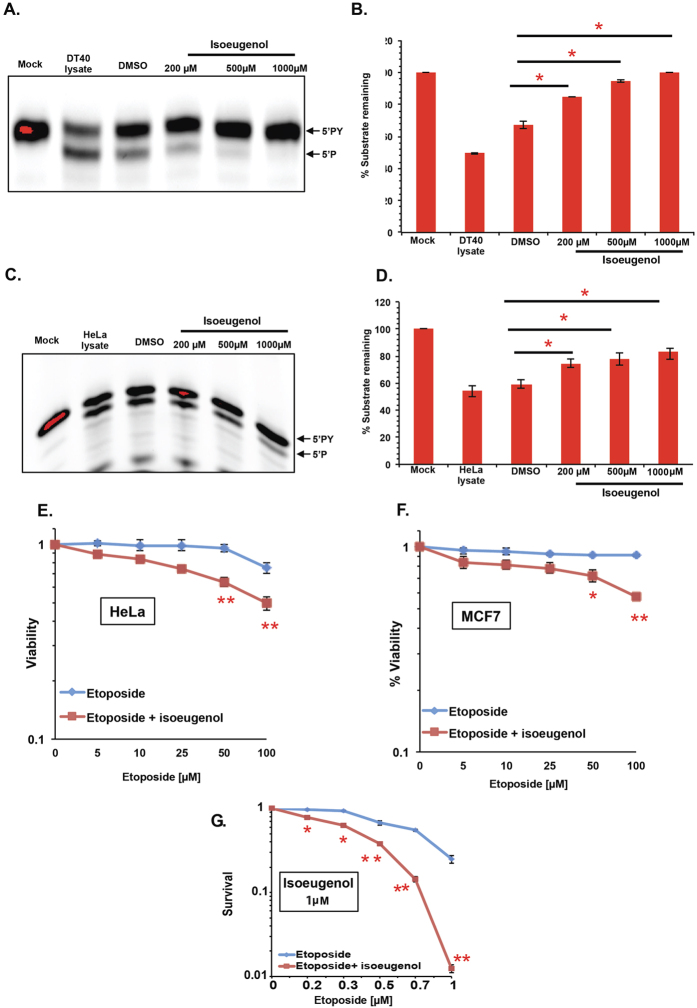
Isoeugenol inhibits TDP2 catalytic activity *in vitro* and potentiates etoposide cytotoxic effect *in vivo*. (**A**) Wild-type DT40 cell lysate was incubated with DMSO or with the indicated concentrations of isoeugenol. Reaction products were resolved by denaturing PAGE and analysed by fluorescence imaging. Arrows point at the position of substrate (PY) and cleaved product (P). (**B**) The bands from A were quantified using ChemiDoc™ MP Imaging System software and the average % substrate remaining of three independent experiments ± s.e.m is shown. Asterisks denote statistical difference (p < 0.01, 1000 μM and 500 μM; p < 0.05, 200 μM) using t-test between DMSO and each concentration. (**C**) HeLa cell lysate was incubated with DMSO or with the indicated concentrations of isoeugenol and reaction products analysed as described in **A,B**. (**D**) The bands were quantified using ChemiDoc™ MP Imaging System software and the average of % substrate remaining for three independent experiments ± s.e.m is shown. Asterisks denote statistical difference (p < 0.01, 1000 μM; p < 0.05, 500 μM and 200 μM) using t-test between DMSO and each concentration. Viability of HeLa (**E**) and MCF-7 (**F**) cells was compared using the indicated doses of etoposide (sub-lethal: 50, 25, 10, and 5 μM; lethal: 100 μM) in the presence and absence of sub-lethal doses of isoeugenol (200 μM and 400 μM for Hela and MCF-7; resp.). Results are represented on a semi-log graph and present the average of 3 biological replicates ± s.e.m. Asterisks denote statistical difference (p < 0.001, p < 0.05 at 50 μM for HeLa and MCF-7, resp.; p < 0.01 at 100 μM for both cell lines) using t-test. (**G**) HeLa cells were treated with the indicated doses of etoposide in the absence or presence of 1 μM isoeugenol for 10 days and the number of surviving colonies was counted and presented as % survival. Data are the average of 3 biological replicates ± s.e.m. Asterisks denote statistically significant difference between etoposide alone and etoposide in combination with isoeugenol (P < 0.01, 0.001, 0.01, 0.05 and 0.001 for doses 1, 0.7, 0.5, 0.3, 0.2 μM; resp.).

We previously reported that TDP2 could protect from TOP1-induced damage in the absence of TDP1^18^. Consequently, we examined whether or not isoeugenol would inhibit TDP2 activity, potentially providing a mechanistic insight explaining our data. Whole cell lysate from wild-type DT40 was employed as the source for TDP2. Incubation of 5′-phosphotyrosine substrate with increasing concentrations of isoeugenol revealed a dose-dependent reduction in the conversion of 5′-PY to 5′P, which is specific for TDP2 activity (Fig. 4A). Whilst incubation with extract alone led to ~50% processing, co-addition of 200 μM isoeugenol suppressed TDP2 activity resulting in ~34% processing (Fig. 4B). Increasing the concentration of isoeugenol to 500 μM and 1000 μM showed a corresponding increase in the inhibition of TDP2 activity reaching as little as 6% or no conversion, respectively. The putative TDP2 inhibitory effect of isoeugenol was not due to the vehicle in which it was dissolved (DMSO) since we kept the concentration of DMSO constant in all control and test conditions. These observations suggest that isoeugenol inhibits chicken TDP2 catalytic activity at high doses. To test if this is an epiphenomenon for chicken DT40 or is also true in human cells, we repeated the experiments using HeLa cell lysates (Fig. 4C). Consistent with its TDP2 inhibitory activity, isoeugenol also inhibited human TDP2 but less efficiently than chicken TDP2 with only 20% inhibition at the highest isoeugenol dose examined (1000 μM). If isoeugenol inhibits TDP2 activity we reasoned that it would sensitize human cells to TOP2 poisons, which specifically require TDP2 to liberate stalled TOP2 from DNA termini. Incubation with etoposide led to a dose dependent decline in viability of both HeLa and MCF7 cells and the co-addition of isoeugenol resulted in further sensitisation (Fig. 4E,F). At 50 μM etoposide, the viability of HeLa and MCF-7 cells dropped, upon the addition of isoeugenol, from 94.8% to 64.1% and from 91.1% to 72.1%, (P < 0.001, P < 0.05; resp.). At a higher dose of 100 μM etoposide, viability of HeLa cells decreased from 75.5% to 49.6%, and from 91.2% to 56.9% for MCF-7 cells (P < 0.01). Importantly, addition of low micromolar doses of isoeugenol (1 μM) sensitized HeLa cells to all concentrations of etoposide tested, as measured by clonogenic survival assays (Fig. 4G). Together, these findings identify isoeugenol as a promising lead compound with potential TDP2 inhibitory activity.

## Discussion

Drug combination therapy is a promising strategy used in treating complex diseases such as cancer, cardiovascular diseases, and infectious diseases^19^. Synergistic drug combinations are recognized as effective and therapeutically specific^20^. Overcoming toxicity, side effects linked to high doses of single drugs, and drug resistance can be achieved by synergistic combinations of two or more agents. Consequently, this study aimed to achieve a synergistic therapeutic effect and minimize dose through combination between the TOP1 inhibitor CPT and natural compounds. Screening of 41 compounds from natural origin belonging to diverse classes of natural products viz. alkaloids, phenylpropanoids, saponins and terpenes in combination with CPT revealed a synergistic effect between the phenolic ether isoeugenol and CPT against Tdp1−/− DT40 cells. Such a synergistic effect encourages further research and may lead to a new anti-cancer strategy that target a specific class of tumors, such as those that develop resistance to camptothecins, for example colorectal cancers (CRCs). Our screen identified isoeugenol, a phenylpropanoid, as a compound that potentiates CPT cytotoxic effect on cells that lack TDP1. Several studies have shown that phenylpropanoids exhibit different biological activities that include analgesic, anti-inflammatory, and anti-tumor activities^21^,^22^,^23^. Isoeugenol (4-propenyl-2-methoxyphenol) is abundantly present in several plant essential oils and is regularly used in spices, perfumes, and detergents, thus is unlikely to exert toxic effects on humans^24^. It is a structural isomer of eugenol found in clove and cinnamon oil and is mostly recognized medicinally for its local anesthetic effect^25^. Notably, previous studies have shown that eugenol exhibits a topoisomerase II (TOP2) inhibition activity^26^,^27^, however, to the best of our knowledge, there are no studies conducted on isoeugenol. Eugenol was found to display genotoxic activity via TOP2 inhibition, halting cells in the replication phase, causing S-phase arrest and apoptosis. In addition, eugenol upregulates numerous enzymes involved in the base excision repair pathway and E2F family members in addition to its potential synergistic effect with gemcitabine and fluorouracil on HeLa cells^26^.

In an attempt to examine the mechanism through which isoeugenol potentiates CPT cytotoxic effect on TDP1-deficient cells, we have conducted a series of cellular and biochemical experiments to test specific PDB repair pathways. First, the PARP1-TDP1 pathway was assessed. PARP1 directs TDP1 towards the break induced by CPT, where TDP1 cleaves the bond between TOP1 and the DNA 3′-terminus. This is followed by modification in the 3′- and 5′-termini via polynucleotide kinase phosphatase (PNKP), and finally ligation by DNA Ligase III (Lig3α)^28^. Our findings suggest that isoeugenol potentiates CPT cytotoxicity independently of TDP1 for multiple reasons. First, only TDP1 deficient, but not proficient, cells were responsive to isoeugenol. Second, biochemical analyses failed to detect inhibitory effect of isoeugenol on established TDP1 substrates. Third, isoeugenol did not further sensitise CPT-treated cells to the PARP1 inhibitor olaparib. It is possible that isoeugenol targets a parallel nucleolytic pathway or another tyrosyl DNA phosphodiesterase that has been implicated for TOP1-mediated PDB repair, particularly in absence of TDP1. Our data favors the latter possibility since isoeugenol displayed detectable TDP2 inhibitory activity *in vitro* when incubated with cell lysates from Chicken DT40 or HeLa cells. These observations were further supported by a significant potentiation of cytotoxicity inflicted by the TOP2 poison etoposide, which specifically generates TOP2-mediated PDBs for which TDP2 is required for processing.

TDP2′s discovery as a 5′-tyrosyl DNA phosphodiesterase in 2009^29^ has since impelled scientists to search for an inhibitor. It has been proposed that drug resistance to TOP2 inhibitors, such as etoposide, may stem from an acquired or intrinsic overexpression in TDP2, which as discussed earlier guards against the abortive activity of TOP2 during replication and transcription. In 2013, Oglivie *et al*. have managed to uncover two classes of compounds, toxoflavins and deazaflavins, as selective TDP2 inhibitors following a high-throughput screening^30^. More recently, isoquinoline-1,3-diones was also found to selectively inhibit TDP2 at a low micromolar concentration^31^. Isoeugenol exhibits a dose dependent inhibitory activity on TDP2, but not TDP1, albeit at higher concentrations. Notably, vanillin (4-hydroxy-3-methoxybenzaldehyde) a structural analogue for isoeugenol (Supplementary Fig. 5) that only differs in an aldehyde group instead of a prop-1-ene moiety displayed no activity in our assays, suggesting that the presence of an alkenyl group is crucial for biological activity. *In planta*, there exists several other isoeugenol analogues particularly found in spices and medicinal herbs such as eugenol anethole, estragole, safrole, myristicin, and methyl isoeugenol, all of which have yet to be assessed and might provide more potent drug candidates. Finally, We noted that the effect of isoeugenol on Tdp1−/− DT40 cells was more prominent compared to TDP1 depleted human cells. This may reflect residual TDP1 in human cells, due to siRNA knockdown versus genetic disruption in DT40 cells, which is sufficient to mask the requirement for alterative pathways (i.e. TDP2). Alternatively, it could reflect the differential inhibition of chicken and human TDP2 by isoeugenol. In support of the latter possibility, incubation of 500 μM isoeugenol with DT40 cell extract nearly reached full inhibition with ~100% substrate remaining (Fig. 4A,B) versus ~80% for HeLa cell extract (Fig. 4C,D). These observations point at cross species differences, which may be illuminating in future studies aiming at improving the potency of isoeugenol as a candidate for drug discovery.

In summary, we identify isoeugenol as a potentiator of CPT cytotoxicity in a TDP1 dependent manner and suggest that it acts by inhibiting TDP2 activity. Isoeugenol therefore may act as a potential lead compound for developing TDP2 inhibitors. Future structure-activity relationship studies might be warranted to improve potency and shed light on its utility in drug discovery programs.

## Materials and Methods

### Chemicals

Caffeic acid, *p*-Coumaric acid, naringinen, chlorogenic acid, quercetin, isoquercetin, umbelliferone, harmine, luteolin, caffeine, 2,4,4′-trihydroxy chalcone, naringinin-7-glucoside, pseudoephedrine, glycyrrhizin, epicatechin, vanillic acid, gallic acid, β-glycyrrhetenic acid, xanthohumol and amentoflavone were purchased from Sigma Aldrich (St. Louis, MO, USA). Bisabolene, α-ionone, (*Z*)-3-hexenyl acetate, allyl anthranilate, isoeugenol, α-pinene, cuminaldehyde, thymol, caryophyllene oxide, carvone, and methyl disulphide were provided from Bedoukian Research (Danbury, CT, USA). Cynarin, emodin, rutin, and sennoside A were purchased from Chromadex (Wesel, Germany). Khellin was a gift from Prof. Dieter Treutter (Tech Univ. of Munich, Germany). Sarcophine was isolated from *Sarcophyton ehrenbergi* soft coral whereas vitexin and chrysin were isolated from *Passiflora edulis* leaf in Dr. Farag laboratory, Cairo University (Supplementary Fig. 6).

### Cell culture

Tyrosyl-DNA phosphodiesterase 1-knockout DT40 cells (Tdp1−/−) and those complemented with human TDP1 (hTDP1) were cultured at 37 °C and 5% CO2 (NuAire) in RPMI 1640 medium (Lonza) supplemented with 7% FBS (Sigma), 3% chicken serum (Sigma), 1% L-glutamine (Lonza), and 1% penicillin/streptomycin (Lonza). These cell lines have been previously reported and described^18^. HeLa and MCF-7 cells were grown in Dulbeco’s Modified Eagle’s Medium (DMEM) (lonza) supplemented with 10% FBS (Sigma), 1% L glutamine (Lonza), and 1% penicillin/streptomycin (Lonza) at 37 °C and 5% CO2.

### Viability assays and clonogenic survival

The compounds were screened on both DT40 Tdp1−/− and DT40 Tdp1−/− complemented with hTDP1. Cells were seeded into 96-well plates at a density of 3000 cell/well on day 0. The compounds, CPT (Sigma), and other inhibitors were added at varying concentrations in triplicate. On day 2, 20 μl cell-titer blue (Promega) was added and the plate incubated at 37 °C. After 4 hours, the viability was quantified as fluorescence intensity using a microplate reader, FLUOstar Omega (BMG LABTECH). Olaparib (MedKoo Biosciences) and isoeugenol (0.1 and 50 μM, resp.) were co-added along with increasing concentrations of CPT to DT40 cells (2000 cell/well). After a 72-hour incubation, viability was assessed using the cell-titer blue kit as above. HeLa and MCF-7 cells (5000 cell/well) were treated with five doses of etoposide (Sigma) one lethal (100 μM) and four sub-lethal doses (50, 25, 10, and 5 μM)in the presence or absence of sub-lethal doses (200 and 400 μM for HeLa, MCF-7; resp.) of isoeugenol in triplicate. Treated cells were incubated at 37 °C for 48 h and cell viability measured using the cell-titer blue. Two hundred HeLa cells were seeded in 6 cm dishes at 37 °C overnight. Cells were treated with DMSO (Sigma) or indicated doses of etoposide (1, 0.7, 0.5, 0.3, 0.2 µM) in the presence or absence of isoeugenol (1 μM). Cells were allowed to grow for 10 days. Visible colonies were stained and survival calculated a described below. MCF-7 cells were transfected using siPORT™ NeoFX™ (Invitrogen) transfection reagent with siRNA at a final concentration of 100 nM. The siRNA sequences that were used to target TDP1 are 5′-CUAGACAGUUUCAAAGUGA-3′ 5′-UCACUUUGAAACUGUCUAG-3′, 5′-GACCAUAUCUAGUAGUGAU-3′ 5′-AUCACUACUAGAUAUGGUC-3′, 5′-UCAGUUACUUGAUGGCUUA-3′ 5′-UAAGCCAUCAAGUAACUGA-3′, and 5′-GGAGUUAAGCCAAAGUAUA-3′ 5′-UAUACUUUGGCUUAACUCC-3′, and for scrambled siRNA control, 5′-GCGCGCUUUGUAGGAUUCG-3′ 5′-CGAAUCCUACAAAGCGCGC-3′ was used. Cells were incubated at 37 °C for 24 h then the media replaced with complete media and incubated for an additional 24 h. One hundred MCF-7 cells were seeded in 6 cm dishes at 37 °C overnight. Then cells were treated with DMSO (Sigma) or indicated doses of CPT in the presence or absence of isoeugenol (4 μM) in triplicate. Cells were allowed to grow for 14 days. Colonies were fixed with 75% methanol and stained with 1% Giemsa stain. The surviving fraction was calculated as ((colonies counted/total cells seeded)^treated^/(colonies counted/total cells seeded)^untreated^).

### Western Blot

Cells were lysed in lysis buffer (10 mmol/L Tris-HCl (pH 7.4), 150 mmol/L NaCl, 1 mmol/L EDTA, 0.5% NP40, 50 mmol/L NaF, 1 mmol/L phenylmethylsulfonyl fluoride, and 1 mmol/L Na3 VO4). Total protein concentration was determined using Bradford assay, and equal amounts of total protein were then boiled at 95 °C for 5 min with SDS-polyacrylamide gel electrophoresis (PAGE) sample buffer (25% glycerol, 31.2 ml 0.25 M Tris-HCl (pH 6.8), 7.5 ml 10% SDS, 0.3 M DTT and a dash of bromophenol blue/100 ml) and separated by 11% SDS-PAGE. Separated proteins were then transferred onto apolyvinylidene difluoride (PVDF) membranes (Millipore) following standard methods. The membranes were incubated in blocking solution (2% non-fat milk in PBS) for 1 h, further incubated with primary antibodies (mouse monoclonal anti-TDP1 (Santa Cruze; cat no. Sc-365674) at a dilution of 1:500 and anti-human beta actin at a dilution of 1:1000) overnight at 4 °C, and then incubated with a secondary antibody (anti-mouse horseradish peroxidase, (HRP)-linked IgG antibody at a dilution of 1:1000 for TDP1 and beta actin) (Invitrogen) for 1 h at room temperature. After 3 × 10-min washes in T-PBS (0.1% Tween-20 in PBS) the membranes were developed with enhanced chemiluminescence detection reagent, clarity™ western ECL blotting substrate (Biorad). Signal on membranes was obtained using ChemiDoc™ MP Imaging System (Biorad).

### TDPs activity assay: Gel-based assay

The *in vitro* 3′-tyrosyl-DNA phosphodiesterase 1 and 2 inhibitory activity of compound 14 (200 μM, 500 μM, and 1000 μM) was determined using a gel-based 3′-tyrosyl-DNA phosphodiesterase activity assay. Biochemical assays were performed in 10 μL reaction volumes containing TDP1 reaction buffer (50 mM Tris HCL (pH 7.5), 50 mM KCl, 1 mM DTT, 10 M EDTA, and 100 μg/ml BSA) or TDP2 reaction buffer (50 mM Tris HCL (pH 7.5) 50 mM KCl, 1 mM DTT, 100 μg/ml BSA, and 1 mM MgCl2), cell lysate (14 **≈ **16 μg, TDP1; 14 μg TDP2), and 50 nM Cy5.5-labelled substrate oligomer 5′-(Cy5.5)GATCTAAAAGACT(pY)-3′ (Midland Certified Reagent Company, Texas, USA) for TDP1 or 5′-(pY)CATCGTTGCCTACCAT(Cy5)-3′ for TDP2. The reactions progressed at 37 °C for 2 h (TDP1) and 1 h (TDP2) and were quenched with 10 μL loading buffer (44% deionized formamide, 2.25 mM Tris-borate, 0.05 mM EDTA, 0.01% xylene cyanol, and 1% bromophenol blue). Samples were then heated at 90 °C for 10 min prior to separation on a 20% urea SequaGel by gel electrophoresis at 100 V for 30 min. Reaction products were visualized by gel imaging using ChemiDoc™ MP Imaging System (Biorad)^32^.

### Alkaline single-cell agarose gel electrophoresis assay

Cells were plated and incubated at a density of 60,000 cell/well with 50 μM CPT for 30 min, 50 μM of compound 14, or CPT plus compound 14 at 37 °C for 48 h. DNA strand breakage, primarily (SSBs) and alkali-labile sites, were quantified by alkaline comet assays as described in^33^. Briefly, cells were suspended in pre-chilled phosphate buffered saline (PBS) and mixed with equal volume of 1.3% low-gelling-temperature agarose (Sigma, Type VII) maintained at 42 °C. Cell suspension was immediately layered onto pre-chilled frosted glass slides (Fisher), pre-coated with 0.6% agarose. The slides were maintained in the dark at 4 °C until set, and for all further steps. Slides were immersed in pre-chilled lysis buffer (2.5 M NaCl, 10 mM Tris-base, 100 mM EDTA (pH 8.0), 1% Triton X-100, and 10% DMSO; (pH 10.0)) for 1 h, washed with pre-chilled distilled water (2–10 min), and placed for 30 min in pre-chilled alkaline electrophoresis buffer (50 mM NaOH and 1 mM EDTA). Electrophoresis was then conducted at 1 V/cm for 30 min, followed by neutralization in 400 mM Tris- HCl (pH 7.0) for 1 h. Finally, DNA was stained with Sybr Green I nucleic (Sigma) (1:10000, in PBS) for 30 min. Average tail moments from 150 cells per sample were measured using Comet Assay IV software (Perceptive Instruments, UK)^16^.

### Statistical analysis

All statistical parameters were performed using excel version 2013 (Microsoft office). Viability was calculated by dividing the mean of treated cells by the untreated cells. Average tail moments from 150 cells per sample were measured using Comet Assay IV software (Perceptive Instruments, UK). All data are presented as means with conforming SEM of three biological triplicates. Student′s t-test was used to compare cell viability and tail moment between compounds, CPT alone, and combination with different concentrations of CPT. In all tests, the level of significance was set at p < 0.05.

## Additional Information

**How to cite this article**: Elsayed, W. *et al*. Isoeugenol is a selective potentiator of camptothecin cytotoxicity in vertebrate cells lacking TDP1. *Sci. Rep.* **6**, 26626; doi: 10.1038/srep26626 (2016).

## Supplementary Material

Supplementary Information

## Figures and Tables

**Table 1:** A list of the compounds employed in the screen.

Compound	Name	Class	CAS number	Selected sublethal dose [μM]
1	Caffeic acid	Phenolic acid	331–39–5	10
2	Khellin	Chromone	82–02–0	10
3	*p*–Coumaric acid	Phenolic acid	7400–08–0	10
4	Prunetin	Flavonoid	552–59–0	10
5	Allyl anthranilate	Terpenoid	7493–63–2	50
6	Cynarin	Phenolic acid	1182–34–9	1
7	Caffiene	Alkaloid	58–08–2	10
8	Isoquercitrin	Flavonoid	482–35–9	10
9	Umbelliferone	Coumarin	93–35–6	10
10	2,4,4′ trihydroxy chalcone	Flavonoid	961–29–5	5
11	Emodin	Anthraquinone	518–82–1	10
12	Chrysin	Flavonoid	480–40–0	10
13	Sennoside A	Anthraquinone	81–27–6	10
14	Isoeugenol	Phenylpropanoid	97–54–1	50
15	Pseudoephedrine	Alkaloid	90–82–4	10
16	Glycyrrhizin	Saponin	1405–86–3	1
17	Epicatechin	Flavonoid	490–46–0	5
18	Rutin	Flavonoid	153–18–4	5
19	Vanillin	Phenylpropanoid	121–33–5	10
20	Gallic acid	Phenolic acid	149–91–7	1
21	Xanthohumol	Flavonoid	6754–58–1	0.1
22	Glycyrrhetinic acid	Terpenoid	471–53–4	10
23	Naringenin	Flavanoid	67604–48–2	10
24	Harmine	Alkaloid	442–51–3	0.1
25	Luteolin	Flavonoid	491–70–3	10
26	Kaempferol	Flavonoid	520–18–3	10
27	Hesperidin	Flavonoid	520–26–3	10
28	Bisabolene	Terpenoid	17627–44–0	0.01
29	α–ionone	Terpenoid	127–41–3	0.01
30	cis–3–Hexenyl acetate	Terpenoid	3681–71–8	0.01
31	α–Pinene	Terpenoid	80–56–8	50
32	Cuminaldehyde	Phenylpropanoid	122–03–2	50
33	Thymol	Phenylpropanoid	89–83–8	50
34	Caryophyllene oxide	Terpenoid	17627–43–9	50
35	Carvone	Terpenoid	99–49–0	50
36	Dimethyl Disulphide	Sulphur compound	624–92–0	0.01
37	Amentoflavone	Flavonoid	1617–53–4	1
38	Sarcophine	Terpenoid	55038–27–2	0.01
39	Chlorogenic acid	Phenolic acid	327–97–9	10
40	Vitexin	Flavonoid	3681–93–4	10
41	Quercetin	Flavonoid	117–39–5	1
