# Supplementary material for: Isoeugenol is a selective potentiator of camptothecin cytotoxicity in vertebrate cells lacking TDP1
Source: Sci Rep. 2016 May 25;6:26626. doi: 10.1038/srep26626 (PMC4879542; doi:10.1038/srep26626)
Supplement: Supplementary Information [file srep26626-s1.pdf]

Supplementary Figures

Isoeugenol is a selective potentiator of camptothecin cytotoxicity in vertebrate cells lacking TDP1

Waheba Elsayed[1#], Lamia El-Shafie[1#], Mohamed K. Hassan[1,2], Mohamed Farag[3], Sherif F. El-Khamisy[1,4*]

[1]Center for Genomics, Helmy Institute for Medical Sciences, Zewail City for Science and Technology, Giza, Egypt.

[2] Biotechnology Dept., Faculty of Science, Port Said University, Egypt

[3]Department of Pharamcognosy, Faculty of Pharmacy, Cairo University, Cairo, 11562, Egypt

[4]Krebs Institute, University of Sheffield, Sheffield, S10 2TN, UK

[#] Joint first authors

*To whom correspondence should be addressed. Email: s.el-khamisy@sheffield.ac.uk

Supplementary figure 1

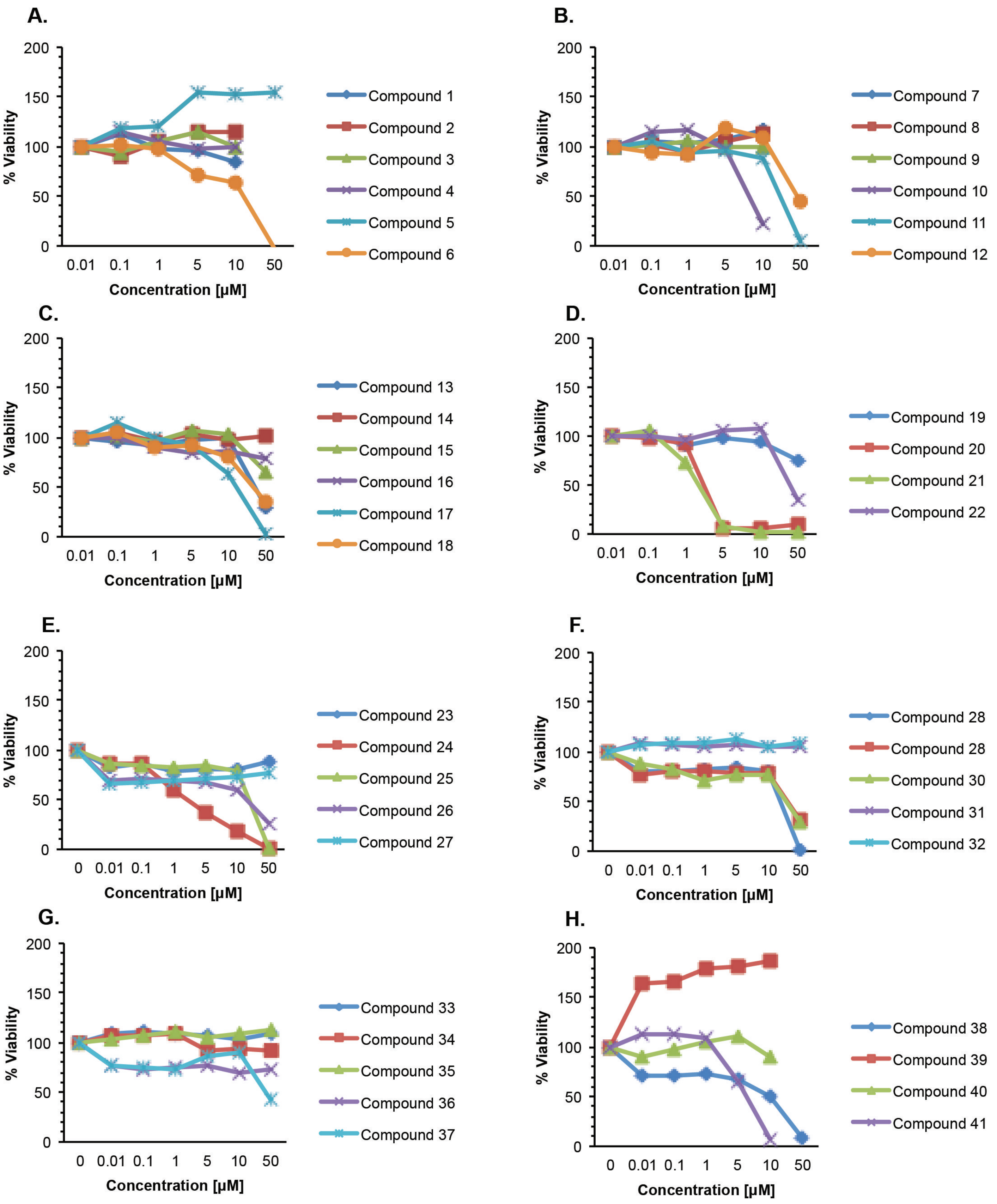

Supplementary figure 1. The cytotoxic effect of library compounds against Tdp1-/- DT40 cells . (A-H) Viability graphs indicating the cytotoxicity of compounds 1 to 41 at six different concentrations (0.01, 0.1, 1, 5, 10, and 50 µM).

Supplementary Figure 2

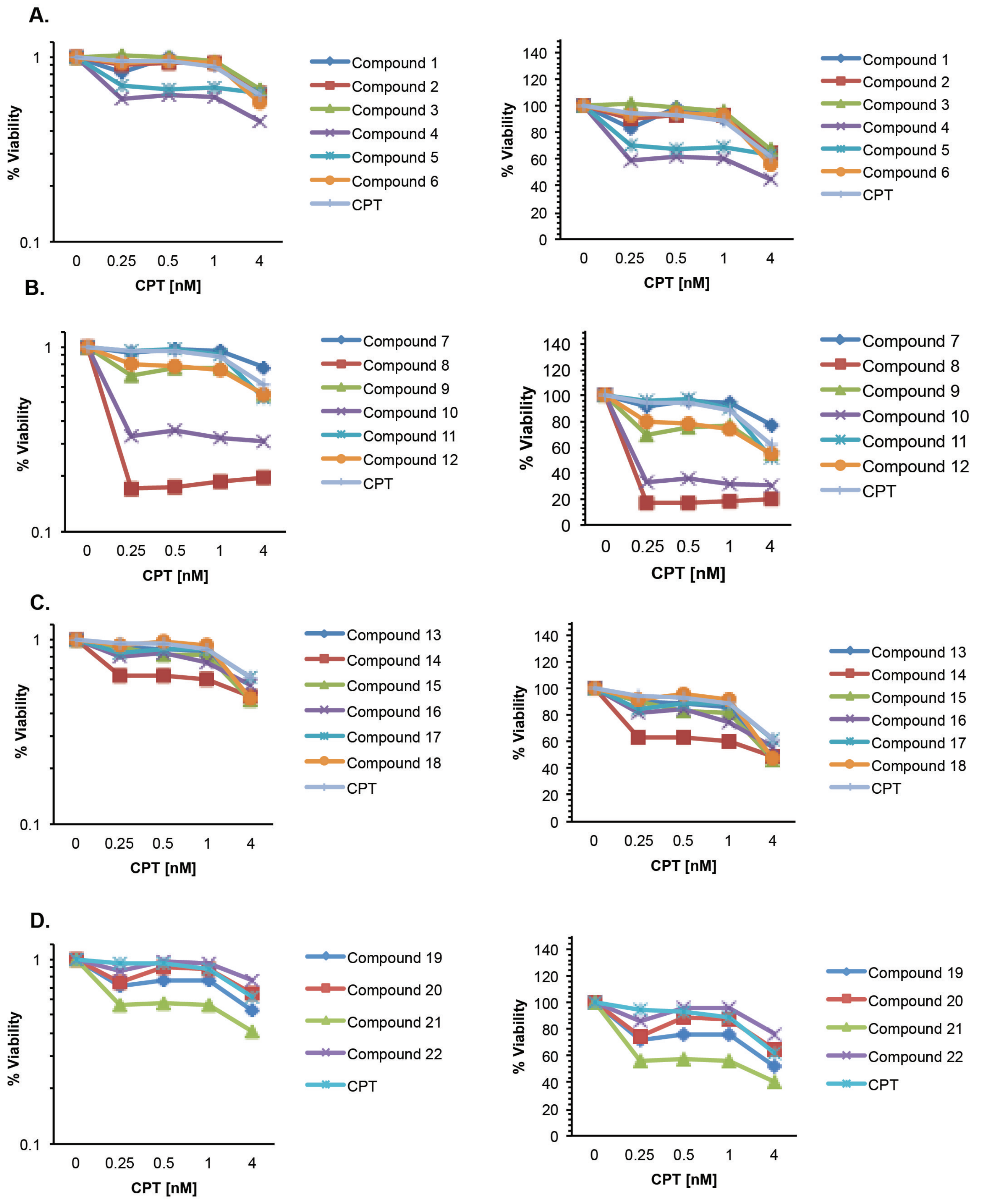

Supplementary Figure 2. (A-D) Primary screen of DT40 TDP1$^{-/-}$ cells against sublethal concentrations of compounds 1 to 22 in combination with CPT at four concentrations (sublethal: 0.25, 0.5, and 1 nM; lethal: 4 nM). ***Left:*** semi log graph; ***right:*** percentage representation.

Supplementary Figure 3

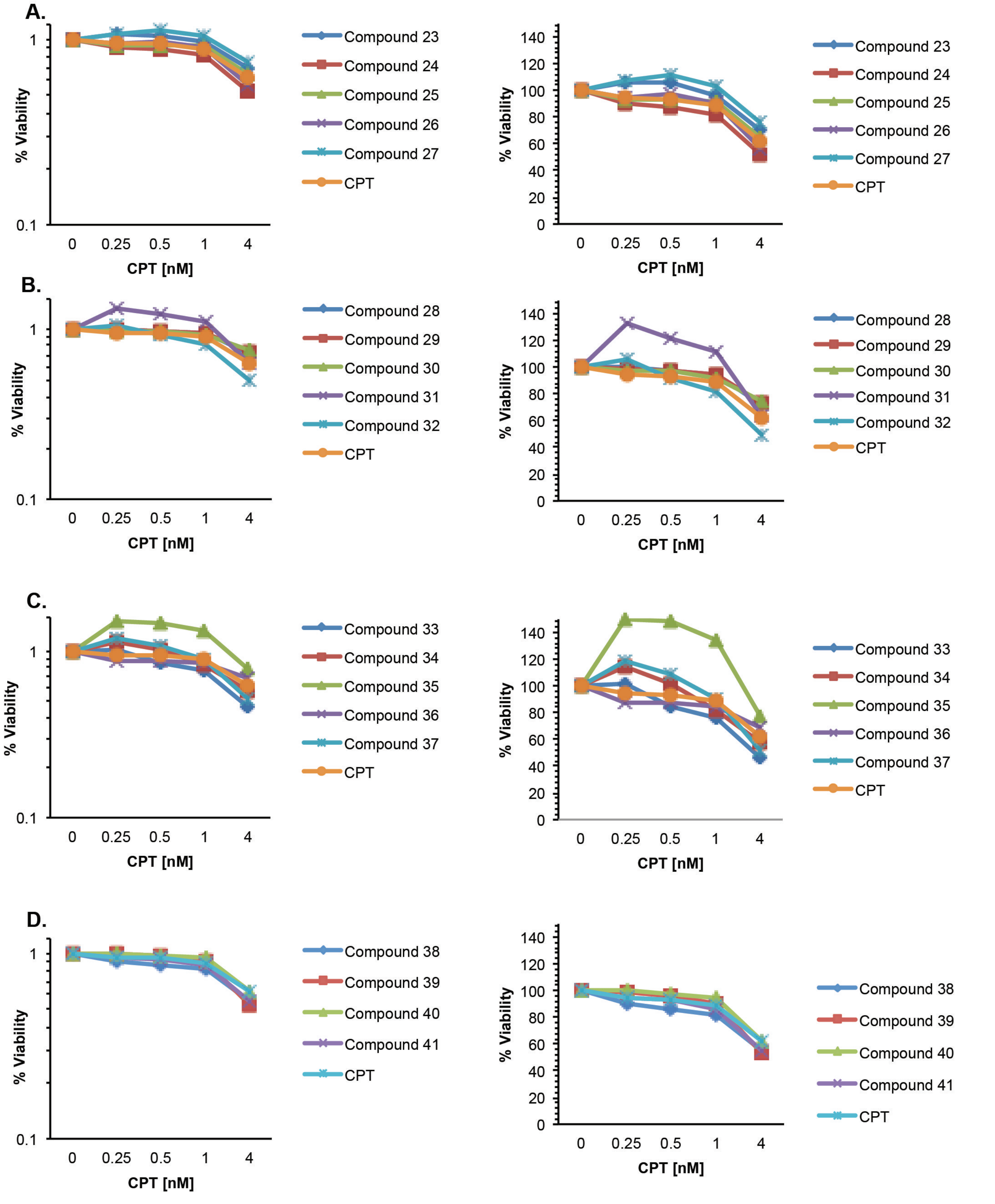

Supplementary Figure 3. (A-D) Primary screen of DT40 Tdp1$^{-/-}$ cells against sublethal concentrations of compounds 23 to 41 in combination with CPT at four concentrations (sublethal: 0.25, 0.5, and 1 nM; lethal: 4 nM). Left: semi log graph; right: percentage representation.

Supplementary Figure 4

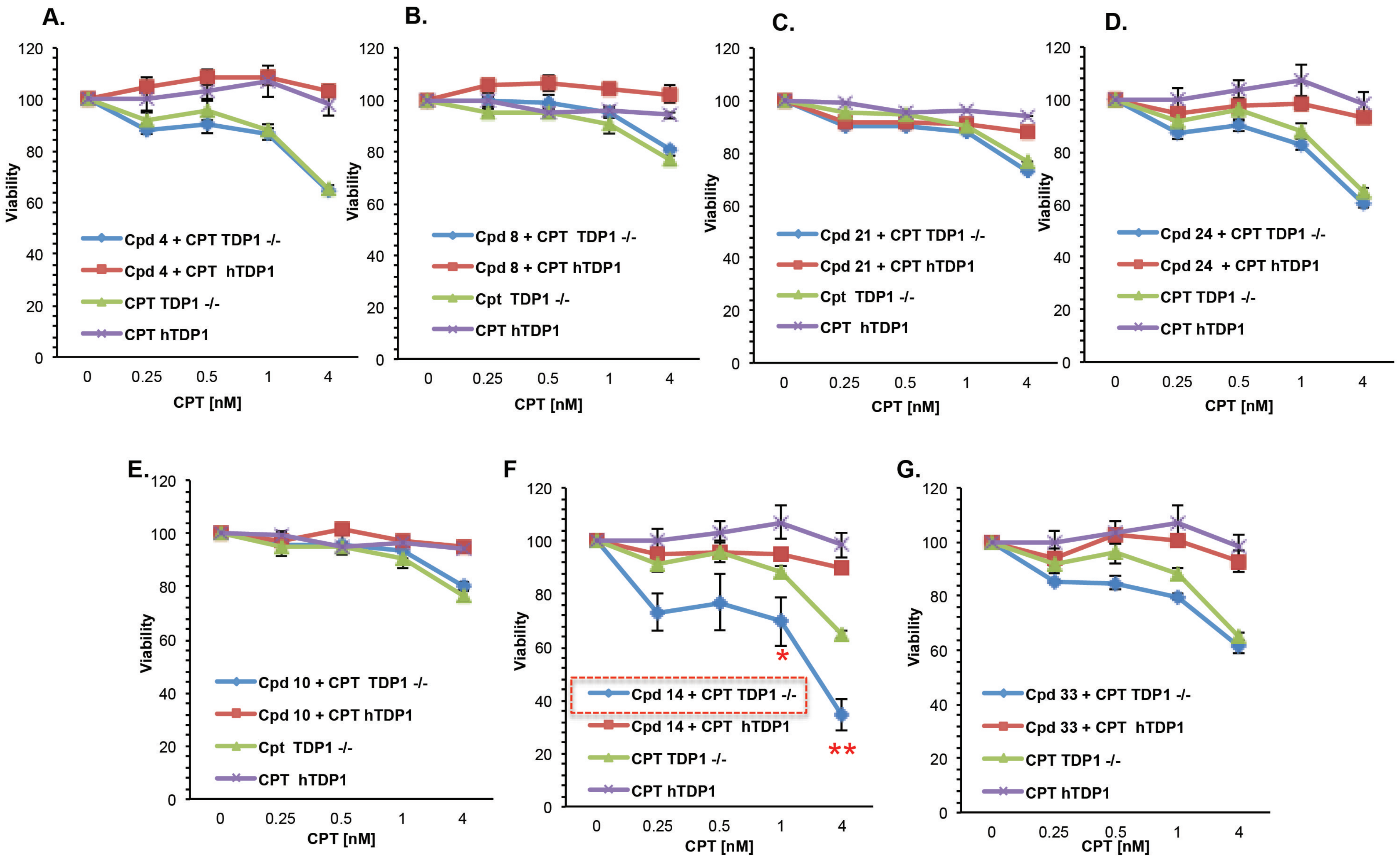

Supplementary Figure 4. Isoeugenol potentiates the cytotoxic effect of CPT in a TDP1-dependent manner. Same data as shown in Figure 1 but the viability is presented as % instead of semi-log scale

Supplementary figure 5

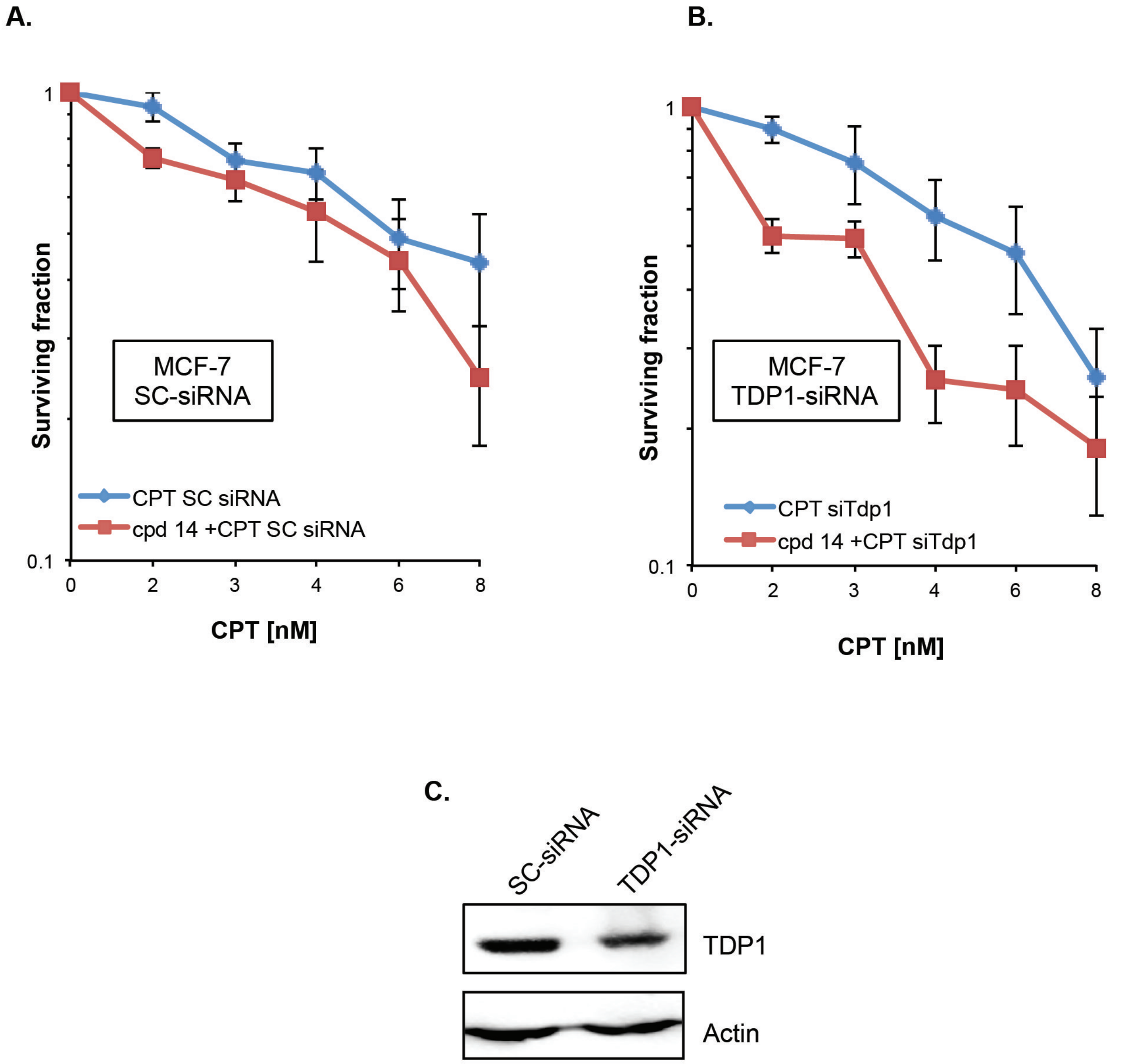

Supplementary figure 5. Isoeugenol potentiates CPT cytotoxicity in TDP1 depleted human cells. MCF-7 cells were treated with a scrambled siRNA "SC-siRNA" (**A**) or TDP1 siRNA "TDP1-siRNA" (**B**) and survival was examined using clonogenic survival assays at the indicated doses of CPT (1 lethal and four sub-lethal: 8nM, 6nM, 4nM, 3nM and 2nM; resp.) in presence or absence of a sub-lethal concentration of compounds 14 (4 μM). Survival was calculated by dividing the average number of colonies on treated plates by the average number of colonies on untreated plates. Data are the average ± s.e.m.. of three biological replicates. **(C)** Cell lysates from A and B were fractioned by SDS-PAGE and analysed by immunoblotting.

Supplementary figure 6

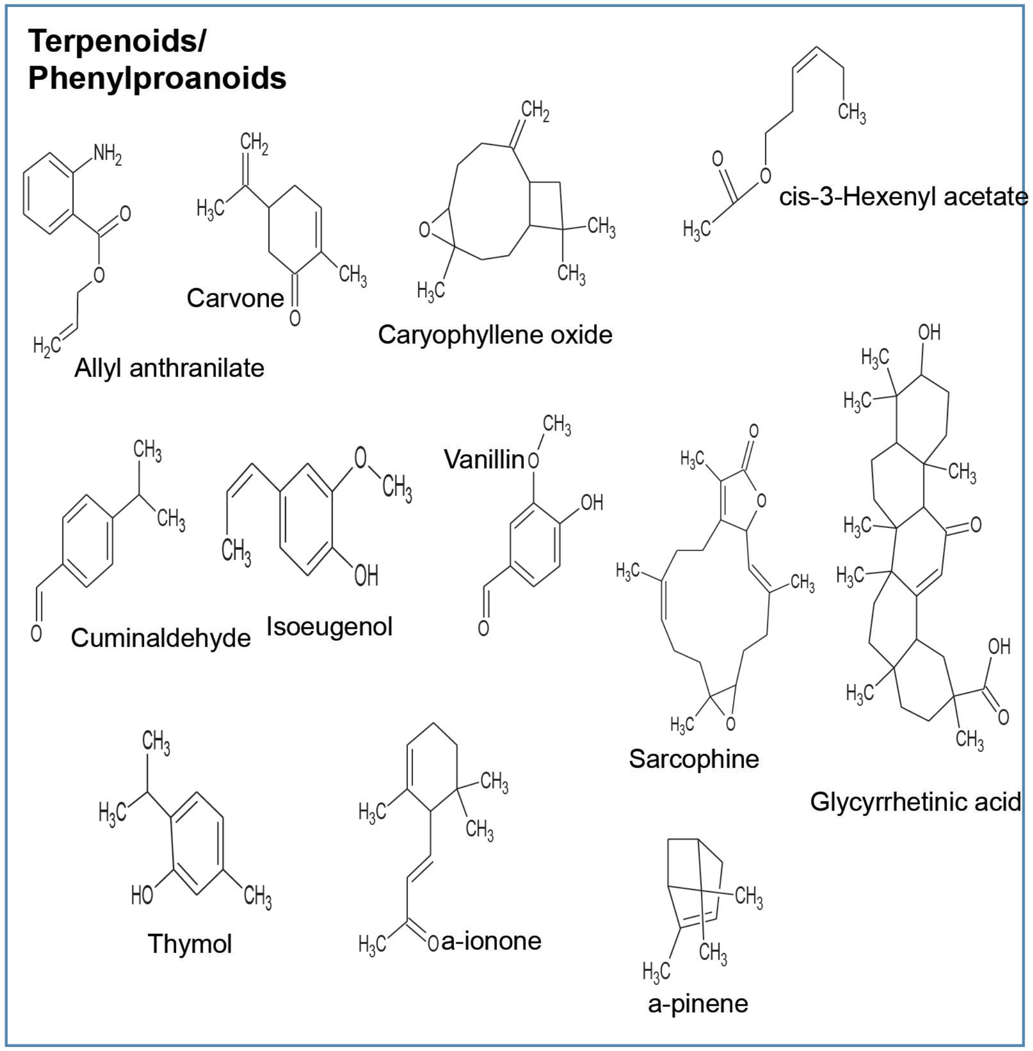

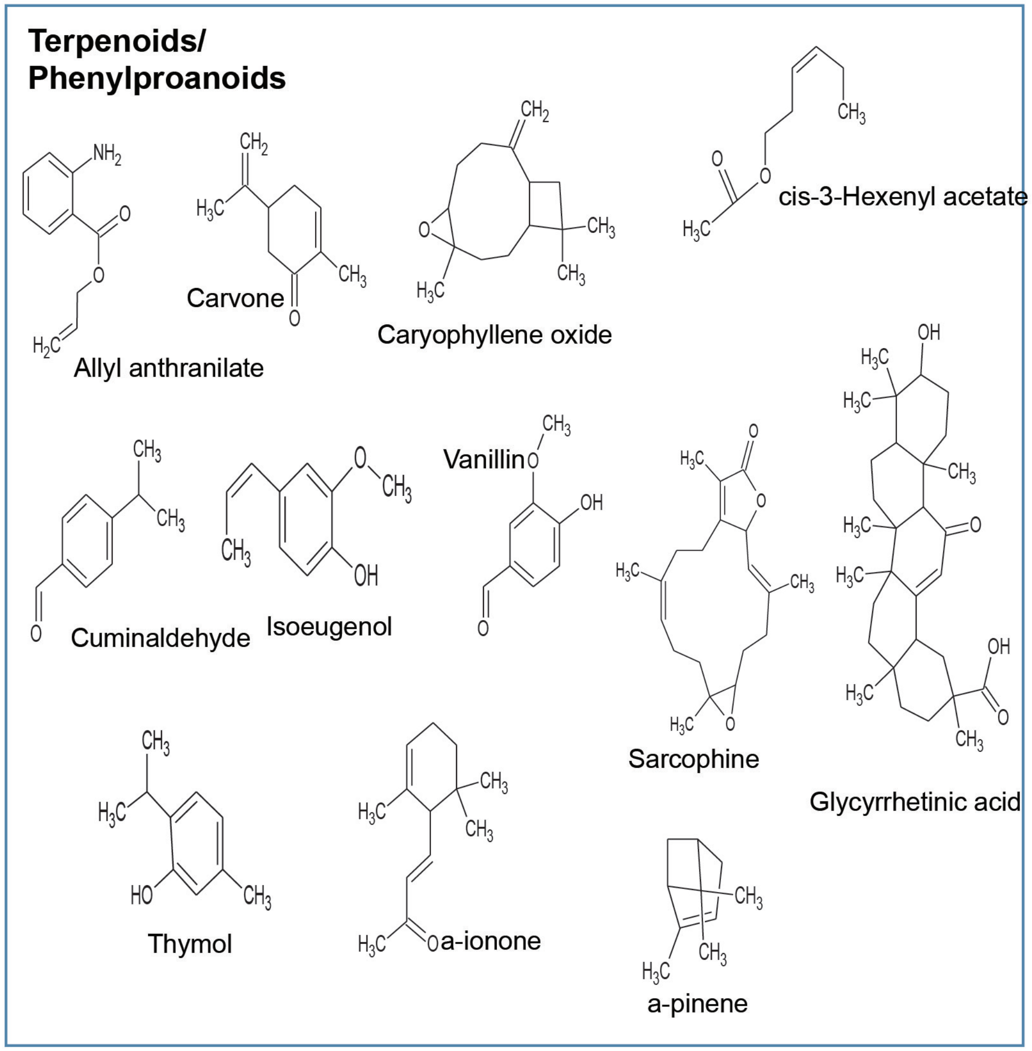

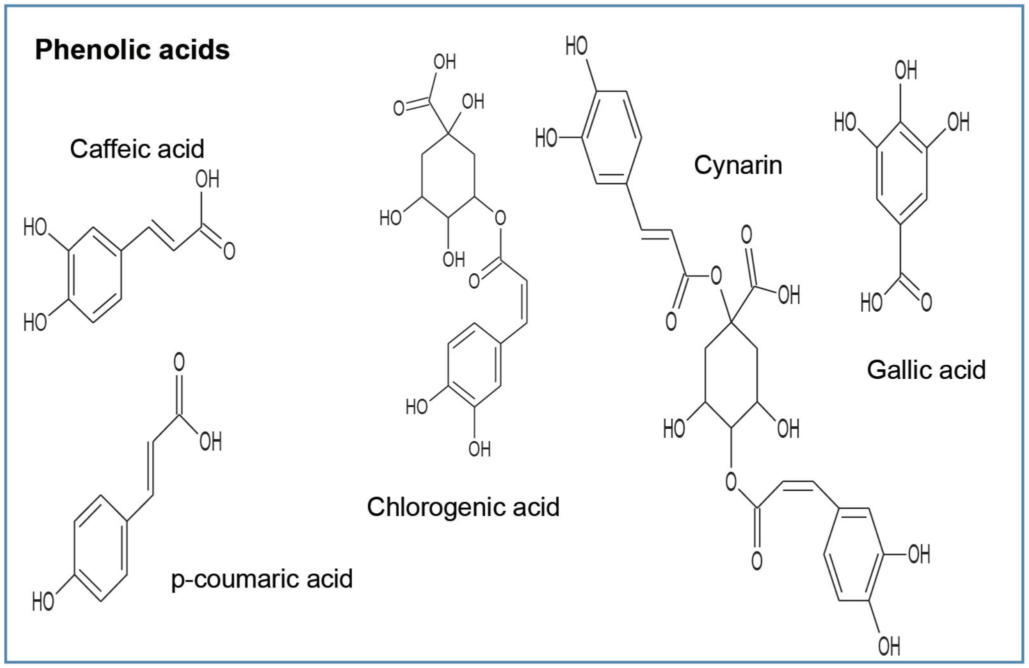

Supplementary Figure 6. Structures of flavonoids, phenolic acids, and Terpenoids/ Phenylproanoids.
